# A Novel Concept of Tissue Micro-Instability as the Underlying Mechanism of Osteophytosis in Human Knee Osteoarthritis

**DOI:** 10.3390/biomedicines14020283

**Published:** 2026-01-27

**Authors:** Alexey Volkov, Vera Lyalina, Gulnara Eshmotova, Natalia Serejnikova, Sofia Petrova, George Airapetov, Evgeniya Parshina, Anton Zalygin, Ekaterina Belitskaya, Vladimir Oleinikov, Anton Bonartsev, Svetlana Borisovskaya, Nikolai Zagorodny, Alexey Prizov

**Affiliations:** 1 St. Alexy Metropolitan of Moscow Central Hospital, 119071 Moscow, Russia; i@bonelab.ru (A.V.);; 2 Department of Pathological Anatomy, Medical Institute, Patrice Lumumba Peoples’ Friendship University of Russia (RUDN University), 117198 Moscow, Russia; 3 Reference Center for Bone Pathology, Patrice Lumumba Peoples’ Friendship University of Russia (RUDN University), 117198 Moscow, Russia; 4 Storozhakov Internal Diseases Department, School of Clinical Medicine, Russian National Research Medical University (Pirogov University), 117513 Moscow, Russia; vera_lyalina@mail.ru (V.L.);; 5 Institute for Regenerative Medicine, Sechenov University, 119991 Moscow, Russia; 6 Faculty of Biology, Lomonosov Moscow State University, 119234 Moscow, Russia; 7 Department of Traumatology and Orthopedics, Medical Institute, Patrice Lumumba Peoples’ Friendship University of Russia (RUDN University), 117198 Moscow, Russiaaprizov@yandex.ru (A.P.); 8 City Clinical Hospital № 31 named after Academician G.M. Savelyeva, 119415 Moscow, Russia; 9 Lebedev Physical Institute of the Russian Academy of Sciences (FIAN), 119991 Moscow, Russia; 10 Shemyakin-Ovchinnikov Institute of Bioorganic Chemistry of the Russian Academy of Sciences (IBCh RAS), 117997 Moscow, Russia

**Keywords:** osteoarthritis, osteophytes, subchondral bone, histology, microcrack, cortical plate, pathogenesis, tissue micro-instability, osteochondral junction

## Abstract

Osteophytes (OPs) are a diagnostic hallmark of osteoarthritis (OA). However, the mechanisms underlying their initiation and their relationship with early subchondral bone changes remain poorly understood. Existing research primarily relies on animal models and late-stage OA tissue, leaving the initial morphological events leading to OP formation unclear. **Background/Objectives**: This study aimed to identify early changes in the subchondral bone as a key trigger for OP initiation in human OA through a comprehensive histological analysis of the subchondral area, including its peripheral regions. **Methods**: We conducted an extensive histological examination of full-section human tibial plateaus, including load-bearing and non-load-bearing compartments, obtained from patients with early and late stages of OA. **Results**: Our data demonstrate that subchondral bone changes, including osteoporosis, osteosclerosis, and microcracks, begin at the pre-chondropathic stage alongside microscopically intact cartilage. We identified a previously undescribed zone on the vertical wall of the tibial condyle (the VEPLS zone), characterized by reduced calcium content in the cortical plate and the persistence of embryonic cartilage, making it morphologically vulnerable. The first event in OP formation is microcracks in the cortical angle and the adjacent subchondral trabecula. These injuries initiate reparative osteogenesis, which, under continuous traumatic load (presumably shear forces due to joint instability), becomes insufficient, leading to cortical angle protrusion and OP formation. OP growth is accompanied by the deformation of the VEPLS zone cortical plate, causing vascular impairment and exacerbating bone weakness. **Conclusions**: Based on our findings, we propose the concept of tissue micro-instability. This concept posits that osteophytosis results from chronic microcracks and failed bone regeneration in vulnerable subchondral structures, induced by joint instability. We define an OP as a pathological outgrowth arising from this tissue micro-instability. Our study highlights the critical role of the peripheral subchondral area, particularly the VEPLS zone, in OA pathogenesis.

## 1. Introduction

The osteoarthritis (OA) involves profound remodeling of the subchondral bone, manifesting as osteoporosis, osteosclerosis, trabecular and cortical micro-fractures, bone marrow edema, subchondral cyst formation, and osteophyte (OP) development [1].

The rapid advancement of regenerative cell-based approaches, particularly those utilizing mesenchymal stromal cells (MSCs) and their derivative exosomes, has greatly deepened the mechanistic understanding of OA pathogenesis at the cellular and molecular levels. MSC-derived exosomes have emerged as highly promising therapeutic candidates, possessing a multifaceted profile that promotes chondrocyte anabolism, modulates inflammation, and critically regulates bone remodeling processes [2,3,4,5]. The molecular mechanisms underlying these effects, including key pathways such as NF-κB and Wnt/β-catenin modulated by MSC-derived exosomes, are well characterized [3]. In contrast, the fundamental morphological and histopathological transformations of the bone during OA progression remain insufficiently explored.

OP are bony outgrowths covered by fibrocartilage, emerging at the peripheral osteochondral junction in both human disease and experimental OA models [6,7,8,9,10,11]. As a defining morphological characteristic of OA, the OP constitutes a key diagnostic criterion for the disorder [12]. No consensus exists regarding the histological classification of OPs. Evidence from animal models and late-stage human OA indicates that OPs progress from mesenchymal-cartilaginous precursors to osteo-cartilaginous structures, with ossification extent correlating with OA severity [13,14]. OPs originate from periosteal and synovial progenitor cells [8,15,16], primarily through endochondral ossification regulated by growth factor signaling [17,18,19].

In advanced OA, OPs are considered as an adaptive response to mechanical overload in degenerative joints, functioning to redistribute stress [20], protect articular cartilage [21,22], and stabilize compromised joints [23,24]. Paradoxically, OPs also emerge during early OA stages [25,26] despite preserved cartilage integrity [8,16], and develop in both load-bearing and non-load-bearing regions, suggesting a generalized articular response [9,27].

Given that cartilage integrity appears insufficient to fully explain OP pathogenesis, mechanistic focus shifts to adjacent tissues. The peripheral osteochondral junction—the precise site of OP development—suggests particular relevance of subchondral cortical and trabecular bone alterations. Early OA stages demonstrate trabecular osteoporosis progressing to late-stage osteosclerosis, with micro-fractures occurring throughout this continuum due to impaired mineralization even in thickened trabeculae. Simultaneously, the subchondral cortical plate undergoes initial thinning followed by subsequent thickening and densification [28,29,30,31].

The causal relationship between these morphological bone changes and OP formation remains unresolved, regardless of whether they drive the process or represent secondary phenomena. Current understanding of OP and subchondral bone complex histomorphology derives predominantly from animal studies [29], particularly post-traumatic OA models [32], which show limited translatability to human age-related OA [14,33]. Human histological investigations remain scarce [14,29], typically focusing on end-stage disease material [29] while neglecting the contextual interplay between OP development and surrounding tissue dynamics [14].

This knowledge gap obscures the initial morphological events in OP pathogenesis. Furthermore, conventional histological approaches concentrate on the horizontally oriented subchondral bone beneath the articular surface, overlooking the peripheral osteochondral junction where OPs actually originate. Furthermore, the hyaline–synovial interface on the vertical condylar surfaces within the joint capsule, which notably contains an extension of the cortical plate, remains particularly under-investigated.

In our study, we aim to achieve the following:

1. Identify early subchondral bone alterations as potential triggers for OP initiation in human OA and delineate their progression throughout OA stages;
2. Perform a comprehensive histological evaluation of the peripheral osteochondral junction, utilizing human tissue from early to late OA;
3. Define the chronological sequence of morphological events culminating in OP development.

## 2. Materials and Methods

### 2.1. Study Cohort

Eighty-five patients were enrolled in this study and categorized into three groups.

Group 1 (pre-OA): Comprised 5 donors (mean age 39.0 ± 9.0 years) with rapidly progressive malignancies (gastric, pancreatic cancer); cadaveric specimens were utilized. Autopsy records confirmed absence of knee joint deformity, macroscopic cartilage alterations, or evidence of prior trauma. Radiographic imaging was not available.

Group 2 (early–moderate OA): Included 12 patients (mean age 69.0 ± 9.0 years) undergoing lower limb amputation for acute popliteal artery thrombosis. Pathological assessment revealed no joint deformity but demonstrated uneven chondropathy with fibrillation, ≤50% cartilage thickness reduction, and moderate meniscal degeneration without traumatic sequelae. Radiographic examination was not performed.

Group 3 (late-stage OA): Consisted of 68 patients (mean age 69.0 ± 9.0 years) receiving total knee arthroplasty for advanced non-traumatic OA. All presented with varus deformity (mean tibiofemoral angle 9.17°) and Kellgren–Lawrence grade 3–4/4 radiological changes [34]. Macroscopic evaluation showed areas of extensive chondropathy with full-thickness cartilage loss, subchondral bone exposure, and degenerative meniscal/ligamentous changes without traumatic evidence.

The study received approval from the Institutional Ethics Committee of Buyanov V.M. Moscow City Clinical Hospital (Protocol #06-07.04.17, 7 April 2017) and complied with Declaration of Helsinki principles. All participants provided informed consent for therapeutic procedures and histological analysis.

### 2.2. Histology

**Figure 1:**
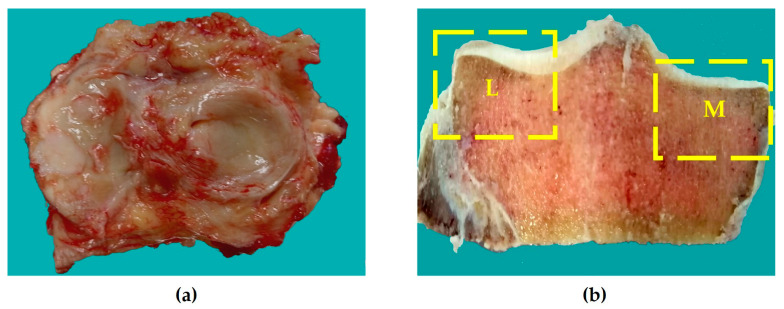
Specimens for histological examination. (**a**) Tibial plateau; (**b**) Regions of interest in the lateral (L) and medial (M) condyles selected for further processing.

Tibial plateau specimens from all patient groups were fixed in 10% neutral buffered formalin (Biovitrum, Moscow, Russia) for 48 h. Following fixation, we demarcated medial and lateral condylar regions (Figure 1) and prepared bone plates using a band saw (BoneSaw J130, Royal Group Co., Tientsin, China). Specimens from the most loaded condylar areas (middle sectors of the medial and lateral compartments) demonstrating pronounced macroscopic changes were selected for analysis. Each sample encompassed the articular surface and underlying condylar structure including the intercondylar region, with any existing OPs preserved in situ.

Select specimens underwent mineralization assessment before decalcification in SoftiDek solution (Biovitrum, Moscow, Russia). Tissue processing followed standard paraffin embedding protocols using isopropyl alcohol (“Biovitrum”, Russia). Histological blocks were sectioned at 5 µm thickness and stained with Hematoxylin and Eosin (Biovitrum, Moscow, Russia), PAS& Fast Green (Sigma-Aldrich, St. Louis, MO, USA), Safranin O and Methyl Green (Biovitrum, Moscow, Russia), and Masson’s trichrome (Biovitrum, Moscow, Russia) to standard protocols. Digital whole-slide imaging was performed using a NanoZoomer S20MD histoscanner (Hamamatsu, Saitama, Japan). Slides displaying maximal pathological severity were selected for histomorphometric evaluation.

A histomorphometric analysis was performed in accordance with ASBMR guidelines [35] to provide an objective assessment of the subchondral cortical plate and the associated trabeculae of the cancellous bone. The parameters measured included Tb. Sp. (trabecular separation) and Ct. Wi. (cortical width) for the subchondral cortical plate and its associated bone trabeculae, both in the subchondral region and within OPs. The longitudinal diameter of the OPs was also measured. The histological stage of OA was determined using the OARSI classification system [36,37]. A decrease in the content of glycosaminoglycans in the cartilage matrix was assessed using a semi-quantitative method based on changes in the intensity of safranin O staining.

### 2.3. Scanning Electron Microscopy

Specimen surfaces were prepared using a Thermo HT252 cryo-microtome (Thermo Scientific, Waltham, MA, USA) at −24 °C followed by distilled water rinsing. Imaging was performed with a Thermo Scientific Quattro S scanning electron microscope (Thermo Scientific, Waltham, MA, USA) operating at 10 kV accelerating voltage with a field emission electron source under environmental mode conditions.

### 2.4. Raman Spectroscopy

Eighty-five patients were enrolled in this study and categorized into three groups. Raman imaging was conducted with an in ViaQontor confocal microscope-spectrometer (Renishaw, Wotton-under-Edge, UK). Spectral acquisition employed a 5× objective, 785 nm laser at full power, and a 1200 lines/mm grating, with 1 s exposure per spectrum. Tissue regions measuring 5 × 3 mm were mapped using a 50 × 30-point grid with 100 µm step resolution, collecting complete spectra across 750–1800 cm^−1^. Analysis targeted the subchondral cortical plate of the tibial plateau and the condylar cortical plate, incorporating adjacent trabecular bone and cartilage regions. Specimens were rinsed with distilled water before analysis to eliminate preservation artifacts.

Samples were mounted in optically transparent Petri dishes with the bone surface immersed under a thin distilled water layer. Data processing utilized WiRE (5.2, Wire Swiss GmbH, Zug, Switzerland) and Spectragryph software (1.2.15, Dr. Friedrich Menges Software-Entwicklung, Oberstdorf, Germany), incorporating cosmic ray removal and baseline correction via built-in WiRE algorithms. Mineralization distribution was visualized through Raman maps based on integrated peak areas in the 960 cm^−1^ region (930–980 cm^−1^), representing hydroxyapatite phosphate vibrations.

Anatomically defined regions of high mineralization—specifically the subchondral plate and condylar cortical plate, excluding trabecular bone—were delineated with transmitted light verification. Regional average spectra were computed and processed in Spectragryph to determine integrated areas for phosphate groups (930–980 cm^−1^, peak 960 cm^−1^), carbonate groups (1050–1100 cm^−1^, peak 1074 cm^−1^), and organic matrix components (1400–1500 cm^−1^, CH_2_ vibrations 1440–1455 cm^−1^) reflecting combined protein/lipid content [38]. Integrated band areas correspond directly to relative abundance of these molecular constituents.

### 2.5. Statistical Analysis

Statistical analysis of morphological parameters was performed using GraphPadPrism software for Windows (9.4.0, GraphPad Software, San Diego, CA, USA). Distribution normality was determined by the Shapiro–Wilk test. The results of semi-quantitative histological scoring with nonparametric distributions (load area, glycosaminoglycan content, cortical plate fractures in the subchondral and VEPLS zones were analyzed by using Mann–Whitney U test. Intergroup differences in quantitative data—including cartilage height, OP diameter, subchondral cortical plate and trabecular thickness, as well as the thickness of the subchondral cortical plate and trabeculae within OPs—were analyzed using unpaired Student’s *t*-tests for normally distributed data. Multiple group comparisons utilized ANOVA or Kruskal–Wallis tests where appropriate. The morphological analysis data were presented as means and standard deviations for quantitative data and as median values and interquartile range for scoring parameters. *p*-values ≤ 0.05 were considered statistically significant.

Scanning electron microscopy and Raman spectroscopy data were processed using GraphPad Prism software for Windows (9.4.0, GraphPad Software, San Diego, CA, USA). Comparative analysis between the tibial subchondral plate and condylar cortical plate was performed using unpaired Student’s *t*-tests for normally distributed data. The results were presented as means and standard deviations.

There are no missing or discarded data in our study. During the statistical analysis, the Z-score method was employed to detect anomalous values in the examined indicators. The statistical analysis employed Pearson correlation in combination with *p*-value assessment to identify and evaluate linear relationships between clinical parameters. Pearson correlation analysis was processed using GraphPadPrism software for Windows (9.4.0, GraphPad Software, San Diego, CA, USA), and identified relationships within tibial plateau parameters. Correlation strength was interpreted as weak (0.01 < r ≤ 0.29), moderate (0.30 < r ≤ 0.69), or strong (0.70 < r ≤ 1.00) for positive associations, with corresponding thresholds for negative correlations. Statistical significance was determined at *p* ≤ 0.05, with further stratification: low (0.01 < *p* ≤ 0.05), moderate (0.001 < *p* ≤ 0.01), and high (*p* ≤ 0.001) significance levels.

## 3. Results

### 3.1. Histological Analysis

A histomorphometric analysis was performed to objectively assess the condition of the cartilage and subchondral cortical plate and associated trabeculae of the spongy bone (Table 1).

#### 3.1.1. Articular Cartilage

All histological evaluations using the OARSI classification demonstrated progressive pathological changes across study groups.

**Figure 2:**
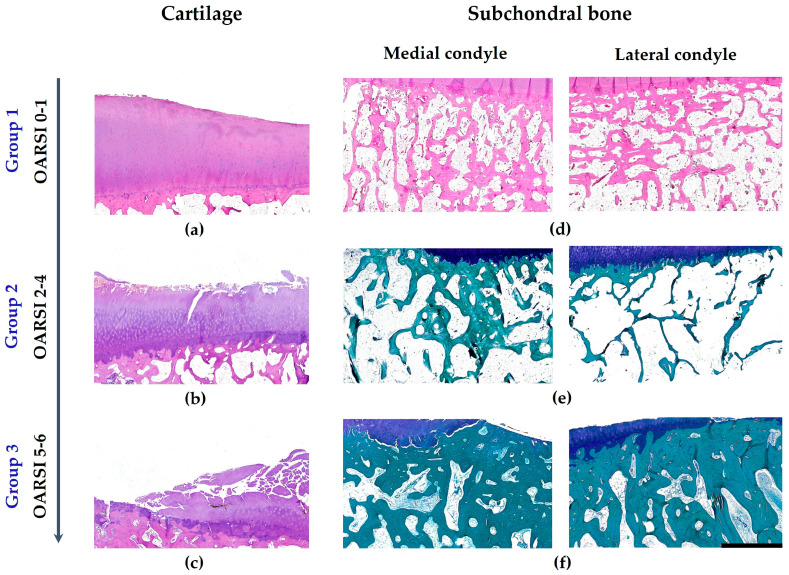
Dynamics of morphological changes in the articular cartilage (**a**–**c**) and subchondral trabecular bone (**d**–**f**) across the study groups. Staining: Hematoxylin and Eosin (**a**–**d**) and PAS-Fast Green (**e**,**f**). Cartilage is colored purple, bone is green, when using PAS-Fast Green staining. Scale bar: 2000 µm.

Group 1 (pre-OA) displayed characteristically thick articular cartilage with preserved surface integrity (Figure 2a). Chondrocytes contained large nuclei without dystrophic alterations and resided within distinct lacunae. The extracellular matrix maintained clear territorial and interterritorial organization, with normal zonal stratification: superficial flattened chondrocytes parallel to the surface, middle zone round chondrocytes in random arrangement, and deep zone columnar formations perpendicular to the articular surface. Minimal focal changes were restricted to the superficial layer. All specimens in this group corresponded to OARSI grade 0–1.

Group 2 (early and moderate OA) exhibited surface villous transformation with superficial fissuring. Notable chondrocyte dystrophy accompanied matrix disintegration and fibrillation (Figure 2b), consistent with OARSI grades 2–4.

Group 3 (late-stage OA) demonstrated advanced cartilage destruction characterized by deep fissuring, zonal chondrocyte dystrophy with cellular abnormalities (hypo- and hypercellularity including clone formation), and profound matrix breakdown. Focal regions showed complete cartilage loss with bone exposure and cyst development (Figure 2c). The medial condyle displayed OARSI grades 5–6 changes, while the lateral condyle maintained OARSI grades 2–4.

#### 3.1.2. Subchondral Trabecular Bone

During early chondropathy (OARSI 0–1) with preserved articular cartilage, the subchondral trabecular bone displayed heterogeneous architecture consistent with Frost’s mechanostat theory [39]. This manifested as osteoporotic regions in low-stress areas and osteosclerotic changes in high-load zones. Specifically, the medial condyle demonstrated osteoporosis peripherally and osteosclerosis centrally, while the lateral condyle showed maximal loading near the intercondylar eminence (Figure 2d). These load-adaptive patterns extended approximately 1 cm deep from the tidemark.

Trabecular dimensions evolved characteristically (Table 1): at stage 0–1, osteoporotic zones measured 92–100 µm versus 150–204 µm in sclerotic regions, compared to the normative range of 100–133 µm [40]. By stages 4–5, these diverged further to 42–96 µm (osteoporotic) and 140–400 µm (sclerotic).

Progressive cartilage degeneration exacerbated these patterns, with original sclerotic zones showing increased trabecular thickness while osteoporotic areas underwent further thinning (Figure 2e,f). Ultimately, at OARSI stage 5–6, osteosclerosis dominated the entire subchondral compartment (Figure 2f).

#### 3.1.3. OPs

**Figure 3:**
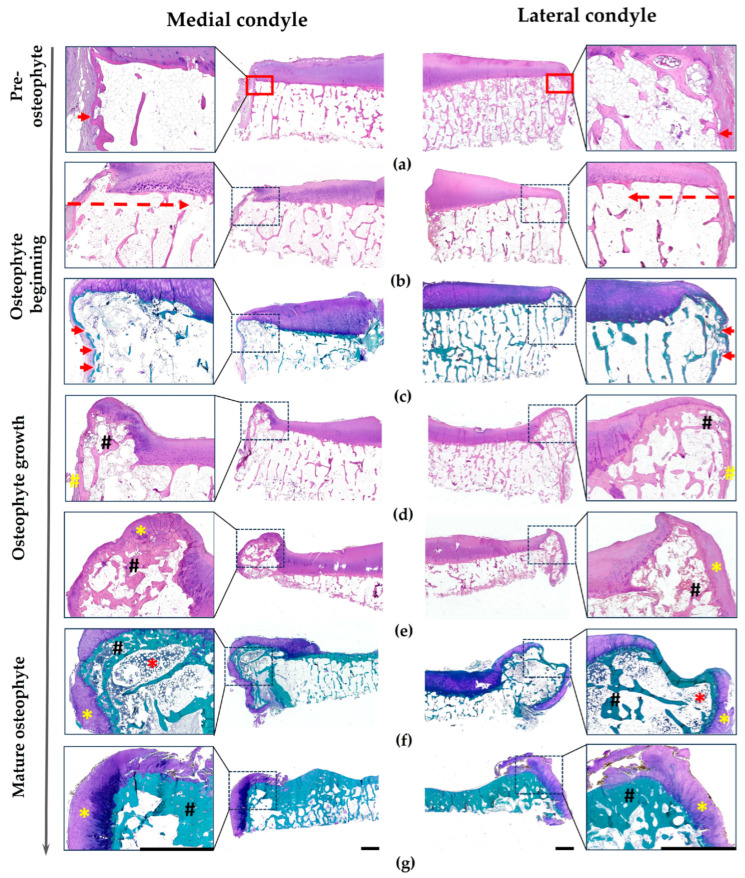
OP development. Legend: red square—transitional angle of the epiphysis; red dashed line—horizontal line of micro-fractures in the subchondral trabecular bone; red arrows indicate micro-fractures of the condylar cortical plate; yellow hatching—periosteum on the OP surface; black hatching—osteogenesis within the OP; yellow asterisk—cartilage on the OP surface; red asterisk—red bone marrow within the OP. Staining: Hematoxylin and Eosin (**a**,**b**,**d**,**e**) and PAS-Fast Green (**c**,**f**,**g**). Cartilage is colored purple, bone is green, when using PAS-Fast Green staining. Scale bar: 2500 µm.

Histological examination of Group 1 specimens with intact hyaline cartilage revealed distinctive features at the transitional angle. This anatomical landmark defines the junction between the tibial plateau and the condylar vertical wall, representing the histological transition from articular hyaline cartilage to the cortical plate of the vertical condylar surface at the periosteum–perichondrium interface (Figure 3a, red square). This transitional region was consistently positioned 2–3 mm from the synovial membrane’s insertion point into the periosteum. Our observations identified a subtle protrusion of the cortical plate at the angular apex, which we termed an “OP bud” (Figure 3a, red square). The base of this nascent structure consistently demonstrated a micro-fracture in the closest peripheral trabecula, and was designated as the “sentinel trabecula” (Figure 3a, red arrow).

Subsequently, the initial fracture of the sentinel trabecula evolved into a clearly defined linear defect. This fracture line then propagated inward through the osteoporotic zone via sequential involvement of adjacent trabeculae, terminating at the boundary with sclerotic bone, beyond which it could no longer be traced. Notably, this linear defect was oriented parallel to the subchondral cortical plate, maintaining a constant distance of 1200–1300 µm from its inferior border.

Beyond the sentinel trabecula fracture, solitary linear micro-fractures developed at the transitional angle’s cortical plate, affecting both its subchondral side (Figure 3b, red dashed line) and vertical side (Figure 3c, red arrows). The latter fracture pathways, initially solitary, later became multiple and consistently coincided with Volkmann’s canals, compromising vascular integrity and resulting in either persistent bone defects or reticulo-fibrous bone formation. While the fracture line became indistinguishable within the maturing OP, the OP itself represented the morphological continuation of this fracture system.

Progressive enlargement of the OP bud resulted in deformation and stretching of the overlying cartilaginous surface, eventually forming a distinct protrusion that remained contained beneath the cartilage, representing early OP development (Figure 3b,c). Subsequently, as the OP matured, its surface retained the hyaline cartilage native to its site of origin. This retained cartilage exhibited a spectrum of changes, ranging from preserved architecture to advanced dystrophic alterations, including matrix fibrillation, progressing to replacement by fibrocartilage. Concurrently, as the OP expanded, its inferior margin gradually incorporated periosteal and cartilaginous elements (fibrous or fibrohyaline) derived from the adjacent vertical condylar wall (Figure 3d–g).

OP expansion involved osteoblast proliferation from the cortical regions of both subchondral and vertical condylar surfaces, progressing through combined primary and endochondral ossification pathways. OP growth patterns were multidirectional, exhibiting lateral, lateromedial, vertical, laterocaudal, and mixed orientations.

Trabecular bone was first detected within OPs at moderate stages of chondropathy (corresponding to OARSI grades 2–3), displaying architectural independence from the subchondral trabecular bone in both thickness and trabecular organization. Mechanical adaptation was evident through the development of load-oriented trabecular arrays responding to asymmetric stress vectors.

Advanced OPs frequently contained red bone marrow within their reticular stroma, contrasting with the epiphyseal and subchondral compartments. This hematopoietic activity coincided with the OP’s structural detachment from the condylar vertical wall, marked by a complete discontinuity of its cortical plate, while its connection to the subchondral cortical bone plate remained intact.

Our analysis revealed no significant correlation between OA stage and OP prevalence, size, or internal trabecular thickness. OP length ranged from 461 to 770 µm at stage 0–1 compared to 397–734 µm at stage 4–5, while trabecular thickness within OPs measured 55–190 µm at stage 0–1 and 56–200 µm at stages 4–5.

#### 3.1.4. The VEPLS Zone

The OP’s inferior margin developed within a distinct anatomical region extending histologically from the articular cartilage–cortical plate junction to the capsular-ligamentous insertion site at the periosteum. This zone is characterized by a high density of periodically arranged Volkmann canals containing vascular bundles.

**Figure 4:**
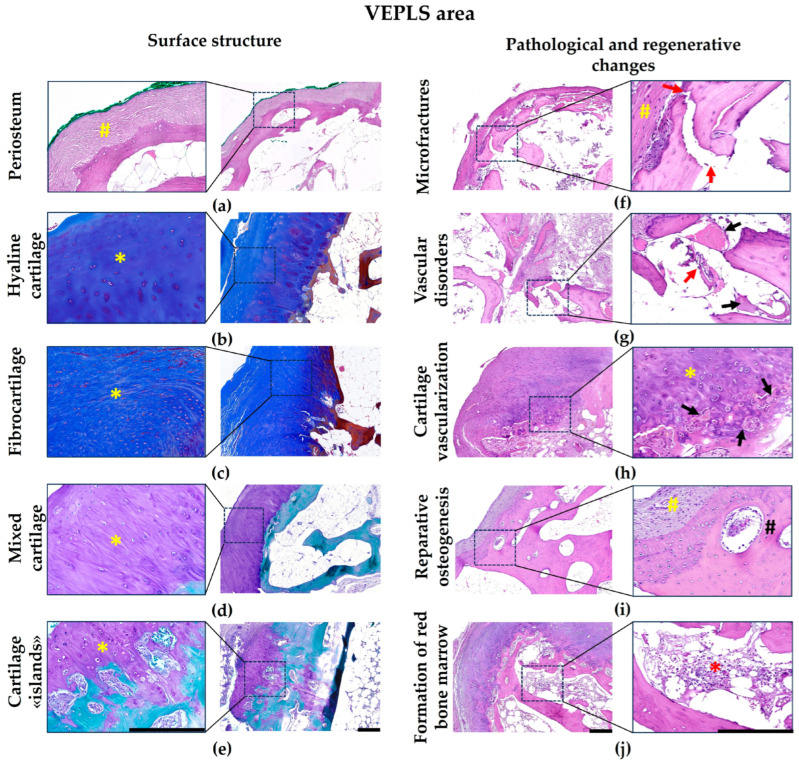
Morphological features of the VEPLS zone. Legend: yellow hatching—periosteum on the OP surface; black hatching—osteogenesis within the OP; yellow asterisk—cartilage on the OP surface; red asterisk—red bone marrow within the OP; red arrows indicate micro-fractures; black arrows indicate blood vessels. Staining: Hematoxylin and Eosin (**a**,**f**–**j**), Masson’s trichrome (**b**,**c**) and PAS-Fast Green (**d**,**e**). Cartilage is colored blue, bone is maroon, when using Masson’s trichrome staining. Cartilage is colored purple, bone is green, when using PAS-Fast Green staining. Scale bar: 250 µm.

While the cortical plate in this region is typically periosteum-covered (Figure 4a), we observed incomplete tissue maturation in 22.5% of cases (18/80 patients), where the surface displayed persistent cartilage of hyaline, fibrous, or mixed fibrohyaline structure (Figure 4b,c). Consequently, the OP’s inferior border demonstrates compositional variability, formed by either periosteum alone or in combination with cartilaginous elements.

The universal pathological features observed in this zone encompassed a triad of interrelated processes. First, horizontal cortical micro-fractures demonstrated reparative osteogenesis from both periosteal and endosteal aspects. Second, a progressive wavelike deformation of the condylar cortical plate developed, emerging in tandem with the initial protrusion of the cortical angle and worsening with its further advancement. This deformation, in turn, directly precipitated the third key feature: damage to the perforating vessels. The vascular injury culminated in interstitial edema and a distinct microcirculatory impairment, manifesting as sludging, stasis, and thrombus formation (Figure 4d,e).

The reparative process followed organized primary osteogenesis patterns without chondroid tissue formation, producing bone structures aligned along load-bearing lines with characteristic epiphyseal vertical orientation. Additional findings included OP micro-fractures and intra-cartilaginous vessels of varying calibers displaying compromised perfusion (Figure 4f).

Due to the distinctive structural characteristics observed, the absence of prior documentation of these changes, and the lack of standardized nomenclature, we have designated this region the VEPLS zone, deriving the acronym from the lead co-authors’ surnames.

### 3.2. Pearson Correlation Analysis

Pearson correlation analysis (Figure S1) revealed several significant relationships in our dataset:

1. Structural interdependence
  A strong positive correlation (r = 0.76, *p* < 0.0001) was observed between the thickness of the subchondral cortical plate and the thickness of the adjacent trabeculae, confirming their structural and functional interdependence as a unified biomechanical system.
2. Ambiguity in the load-response mechanism
  Conflicting correlations within the load-bearing zone demonstrated a dualistic response. Positive correlations were found with cartilage height (r = 0.36, *p* = 0.002) and with the VEPLS-zone cortical fractures (r = 0.37, *p* = 0.002). A negative correlation was observed with trabecular thickness (r = −0.63, *p* < 0.0001). This pattern suggests a complex tissue adaptation mechanism, involving compensatory cartilage thickening concurrent with the development of osteoporosis in unloaded regions.
3. The VEPLS zone as a key link
  Moderate correlations between subchondral cortical fractures and VEPLS-zone cortical fractures (r = 0.34, *p* = 0.005), as well as between the load-bearing zone and VEPLS-zone cortical fractures (r = 0.37, *p* = 0.002), confirm the central role of this zone in transmitting biomechanical stress.
4. Disease progression
  The correlation between the OARSI stage and OP diameter (r = 0.64, *p* < 0.0001) indicates their concurrent progression throughout the disease course.

Thus, Pearson correlation analysis revealed previously unidentified events in OA: a moderate positive relationship between the load-bearing zone of the tibial plateau and cortical fractures in the VEPLS zone, and a relationship of the same magnitude between subchondral cortical fractures and VEPLS zone cortical fractures, serving as evidence linking these processes to OP formation. Furthermore, the analysis revealed strong, direct, and inverse correlations for the load-bearing zone: The correlation was positive with subchondral cortical plate thickness and negative with trabecular thickness. This indicates that increased load leads to greater subchondral cortical plate and trabecular thickness, whereas the absence of load results in localized osteoporosis. A moderately strong negative correlation was also identified between the load-bearing zone and cartilage height in this context.

In summary, the correlation analysis supports the proposed concept of tissue micro-instability, demonstrating the systemic nature of the changes, whereby mechanical loading triggers a cascade of interconnected events across various joint tissues, with the VEPLS zone playing a central role in initiating osteophytogenesis.

### 3.3. Bone Tissue Mineral Composition

**Figure 5:**
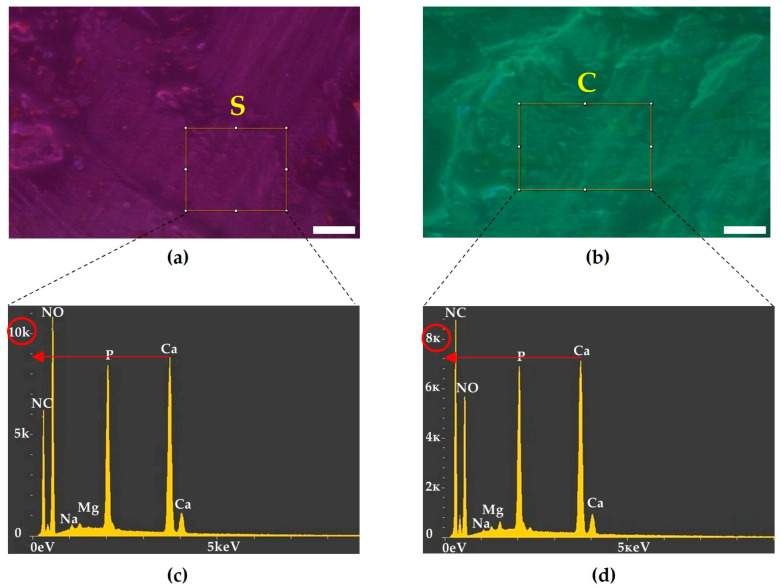
Comparative elemental analysis of the mineral composition in the subchondral versus VEPLS zones of the cortical plate. (**a**) Electron micrograph of the tibial subchondral cortical plate study area (S). (**b**) Electron micrograph of the VEPLS-zone cortical plate study area (C). (**c**) Elemental composition analysis of the tibial subchondral cortical plate. (**d**) Elemental composition analysis of the VEPLS-zone cortical plate. The red arrow points to the relative proportion of calcium ions, circled in red (**c**,**d**). Scale bar: 200 µm.

Elemental analysis via scanning electron microscopy demonstrated a significantly lower relative proportion of calcium ions in the VEPLS-zone cortical plate compared to the subchondral cortical plate (Figure 5).

**Figure 6:**
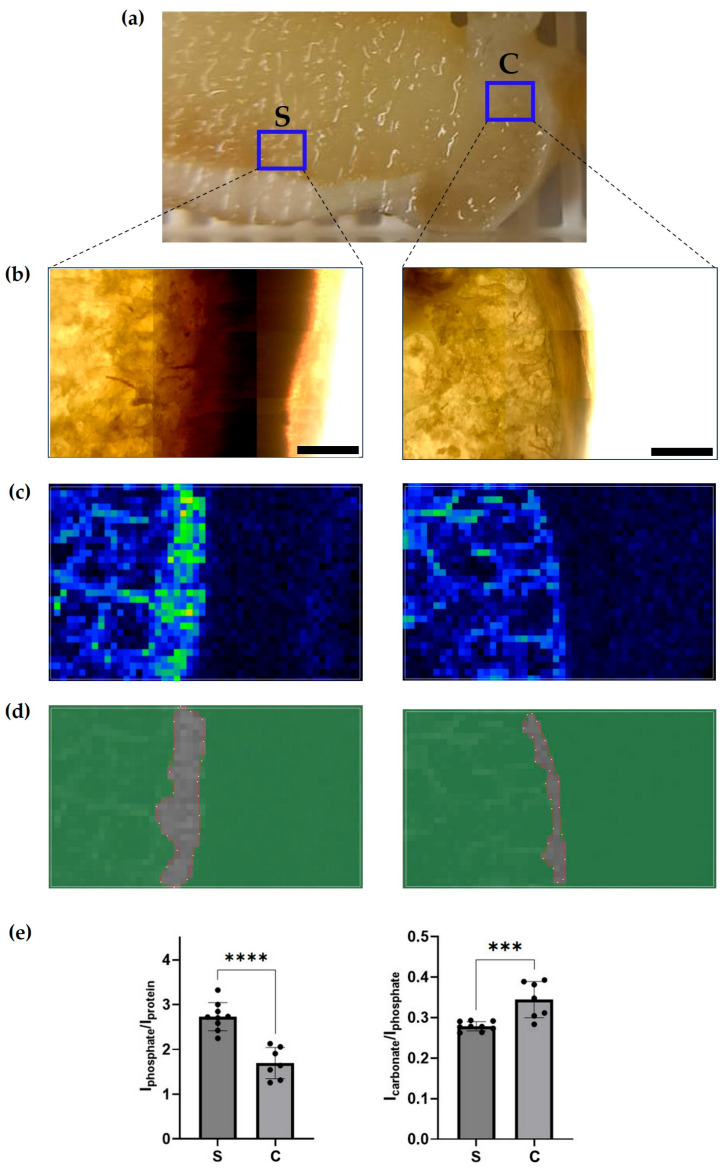
Spectroscopic analysis of the mineral composition in the cortical plate of the tibia. (**a**) Location of the mapped areas: the subchondral zone (S) and the VEPLS zone (C). (**b**) Transmitted light images of the corresponding regions. (**c**) Raman mapping images generated from the spectral integral in the 930–960 cm^−1^ range (phosphate groups). (**d**) Delineated areas of high mineralization used for hydroxyapatite quantification. (**e**) Quantitative analysis showing phosphate groups normalized to the organic matrix (proteins/lipids) and phosphate-to-carbonate ratios. *** *p* < 0.001, **** *p* < 0.0001. Scale bar: 1000 µm.

Additionally, Raman spectroscopy confirmed compositional differences, revealing that highly mineralized areas within the subchondral cortical plate contained greater hydroxyapatite content—reflected by increased phosphate groups—along with reduced carbonate incorporation relative to the VEPLS-zone cortical plate (Figure 6).

## 4. Discussion

While the OP represents a hallmark feature of OA [1], the precise mechanisms governing its formation and its integration with other joint-level pathological processes remain incompletely understood. Current understanding of OA pathomorphology relies heavily on post-traumatic animal models and late-stage human tissue, creating a significant knowledge gap regarding the early morphological events in human disease progression. This gap obscures the temporal sequence of pathological changes [29]. Histological analysis spanning the entire OA spectrum offers a unique opportunity to document disease dynamics and identify correlations between concurrent processes in different joint tissues. To address this, we performed an extensive histological evaluation of complete joint sections from human specimens, analyzing the tibial subchondral bone complex to a depth of 3 cm, along with the associated OPs. Our findings delineate the co-evolution of subchondral bone changes and OP development. This integrative histological assessment represents, to our knowledge, the first study of its kind in human OA.

### 4.1. Early Subchondral Remodeling and a Proposed Refinement of Its Anatomical Definition

Progressive alterations in subchondral bone tissue throughout OA stages increase its susceptibility to fragility and micro-fracture, supporting the “subchondral micro-fracture” concept of OA pathogenesis [28,41,42,43].

The etiology of micro-fractures evolves with disease progression: early-stage OA features trabecular osteoporosis and cortical plate thinning [44,45], while late-stage disease demonstrates impaired mineralization despite trabecular and cortical thickening [46,47,48,49]. Critically, our analysis reveals that subchondral bone changes commence during the pre-chondropathic phase, despite histologically intact articular cartilage. This early remodeling exhibits load-dependent regional specialization, with osteosclerosis developing in high-stress areas and osteoporosis in low-stress regions. These pathological alterations extend approximately 2 cm deep from the subchondral cortical plate’s inferior margin, corresponding anatomically to the synovial–capsular attachment, or the functional joint boundary in the tibia.

This consistent depth distribution warrants refinement of subchondral bone definition. While conventional descriptions characterize subchondral bone as “the bony components lying distal to calcified cartilage” [41,50,51,52], this lacks specific dimensional parameters. We propose redefining the subchondral bone as the architecturally complex bone compartment demonstrating the most significant pathomorphological changes. In the tibial knee compartment, this constitutes the region spanning from the tidemark to the synovial–capsular junction, forming a structurally integrated unit approximately 2 cm in thickness.

### 4.2. Early Structural Failure and an Anatomical Oversight

The presence of trabecular fractures in osteoporotic regions during pre-chondropathic stages reveals underlying bone fragility that precedes substantial cartilage degradation. This suggests mechanisms preliminary to classic cartilage-driven OA pathogenesis. The fracture morphology—characterized by precise linearity, a horizontal orientation (parallel to the articular surface), and intra-tissue occurrence—is indicative of a shear failure mechanism at the subchondral level. This pattern is characteristic of osteochondral or subchondral insufficiency fractures, where shear forces cause a linear cleavage plane through the trabecular bone [53,54,55,56]. Critically, these initial fractures occur in a peripheral zone of the condyle that is subjected to lower primary compressive stress under normal axial loading, as evidenced by biomechanical models of joint contact mechanics [57]. Their lack of impaction damage makes a primary compressive mechanism unlikely. While compressive loads can generate shear at interfaces, this specific morphological context points more directly to horizontal plane shear loading as the predominant disruptive force. The consistent depth (~2 mm below the tidemark) and termination at the cortical angle defines a novel plane of mechanical vulnerability within the subchondral architecture. These findings redirect attention to the under-investigated cortical plate and provides key morphological support for the concept of tissue micro-instability driven by joint-level shear. However, looking ahead, we emphasize that this interpretation, while grounded in histopathology, requires direct experimental biomechanical validation.

The pivotal role of the cortical plate in this proposed mechanism necessitates a closer examination of its anatomy and the current state of knowledge. Despite its potential significance, the cortical plate has received substantially less research attention compared to trabecular bone [29]. Current research on the tibial condylar cortical plate predominantly focuses on its horizontally oriented, strictly subchondral portion [29], which is primarily examined in the context of cortico–chondral interactions [41,49,52,58]. Meanwhile, the subchondral cortical plate curves at the condylar edge forming the cortical angle and extends along the vertical condylar wall, thus becoming the condylar cortical plate. This critical transition zone, along with the condylar portion of the cortical plate, remains largely neglected.

This anatomical region lacks formal designation in current classification systems [59]. To address this terminological gap, we have designated the area on the vertical condylar surface between the plateau’s peripheral edge and the synovial–capsular attachment as the VEPLS zone, establishing a necessary anatomical reference for discussing its significant role in OA pathogenesis.

### 4.3. Structural Vulnerability of the VEPLS Zone

Our findings establish the VEPLS zone as a critically involved region in the progression of subchondral changes, based on distinct morphological characteristics.

Quantitative analysis demonstrates significantly reduced calcium content in the VEPLS zone cortical plate compared to the conventional subchondral cortical plate. This indicates that early OA involves not merely cortical thinning but a fundamental heterogeneity in material composition and mechanical competence. Furthermore, we identified persistent cartilage islands within the VEPLS zone across multiple specimens, presenting as hyaline, hyaline–fibrous, or fibrous cartilage. Since the embryonic epiphysis is entirely cartilaginous and normally undergoes complete ossification [60,61], these findings suggest incomplete postnatal maturation of the condylar cortical plate. This developmental anomaly likely represents an additional factor compromising structural integrity.

The convergence of reduced mineralization and persistent cartilaginous foci creates a morphologically vulnerable region, providing a compelling explanation for the preferential initiation of OP formation at this specific anatomical site.

### 4.4. Initial Phase of OP Development

OP formation commences with concurrent fractures in two locations: the cortical plate at the cortical angle (affecting both subchondral and condylar aspects) and the adjacent subchondral trabecula.

These cortical micro-fractures trigger a reparative response characterized by reticulo-fibrous tissue formation. However, this nascent repair tissue lacks sufficient mechanical strength to withstand ongoing stresses. Under continued mechanical loading, potentially augmented by bone marrow pressure, the cortical angle develops a distinct protrusion oriented toward the condyle’s peripheral margin. This evolving structure subsequently demonstrates simultaneous bone proliferation and compromised regeneration, marking the transition to OP formation.

Concurrent with cortical changes, the initial trabecular fracture propagates centrally, sequentially incorporating trabeculae located further inward into a linear defect. This process creates a structurally distinct osteochondral cap consisting of articular cartilage, subchondral cortical plate, approximately 2 mm of trabecular bone, and the VEPLS-zone cortical plate. Notably, this advancing fracture line demonstrates complete absence of regenerative capacity throughout its progression. Multiple factors likely contribute to this failure: pre-existing osteoporotic changes, the inherently limited regenerative potential of trabecular bone due to its lack of periosteum, and the insufficient biomechanical stimulus from micro-fractures to initiate proper regeneration. The continuous extension of the fracture indicates persistent mechanical disruption. We hypothesize that the resulting osteochondral cap undergoes constant shearing micromotion in the horizontal plane, creating a self-perpetuating cycle of mechanical stress that both propagates micro-fractures and prevents their healing.

Consequently, while cortical and trabecular components follow different pathological progression trajectories after initial micro-fracture, they share a common pathway of failed regeneration.

### 4.5. Concurrent Vascular Pathology in the VEPLS Zone

Progressive deformation of the VEPLS-zone cortical plate during OP growth induces significant vascular compromise. While subchondral vascular alterations are recognized features of OA pathogenesis [52,62]—including angiogenesis [63,64], vascular penetration across the osteochondral junction [49,65], venous stasis with impaired arterial inflow [66], and bone marrow lesion formation [62,67,68]—our findings reveal a previously uncharacterized vascular pathology.

We identify mechanical compression of perforating epiphyseal vessels as a novel mechanism of vascular impairment. The undulating deformation of the VEPLS-zone cortical plate during OP development directly compromises these vessels, resulting in circulatory disturbances characterized by stasis and combined arterial–venous congestion. This vascular compromise likely contributes to trabecular weakening through impaired perfusion of the epiphyseal bone.

### 4.6. The Initiating Traumatic Force and Its Origin

The mechanical force responsible for initiating and perpetuating the observed damage appears to be shear loading resulting from joint instability. The horizontal propagation of the trabecular fractures particularly supports this mechanism.

Joint instability, a well-established factor in OA pathogenesis [33,69,70], stems primarily from dysfunction of soft skeletal structures. The current paradigm attributes this to weakness in periarticular muscles [71,72,73,74,75,76], disrupted muscle–ligament interactions [77], impaired proprioception [76,78], and ligamentous insufficiency due to previous trauma [69], dysplasia [70], or age-related degeneration [76,79,80]. While age-related changes in knee ligaments have been documented, research has focused predominantly on the cruciate ligaments [81,82,83,84], with comparatively less attention given to collateral ligaments [85,86]. Nevertheless, degenerative processes undoubtedly affect the entire ligamentous complex [82].

Although our study did not directly assess ligamentous integrity or document trauma history, the observed pathology suggests that age-related connective tissue degeneration represents a plausible initiating factor. This perspective aligns with emerging views prioritizing ligamentous changes in early OA pathogenesis [87,88]. We therefore propose that ligamentous laxity—among the earliest age-related alterations in joint structures—generates the shear forces responsible for cortical angle protrusion and subsequent linear trabecular fractures.

OP formation as a bony proliferation initiates presumably from periosteal irritation [16] triggered by recurrent micro-fractures. The combined effect of persistent mechanical stress within the subchondral bone and periosteal irritation results in simultaneous endochondral and periosteal ossification with chronically impaired regeneration. This mechanism likely accounts for the documented similarities between OP development and fracture callus formation [15,89]. However, while callus maturation typically concludes within approximately eight weeks, OP growth persists indefinitely [90,91,92], reflecting the continuous nature of the underlying mechanical insult.

The mature OP demonstrates a heterogeneous cartilaginous covering. Fracture sites develop fibrous reparative cartilage, while the OP’s periphery maintains original cartilage structures—hyaline cartilage along the superior aspect [8] and fibrohyaline cartilage or periosteum along the inferior margin. Notably, observed hyaline cartilage foci, previously attributed to neochondrogenesis [7], more likely represent persistent embryonic cartilage remnants. This interpretation is reinforced by our documentation of hyaline cartilage preservation within the VEPL zone at stage “0” chondropathy.

### 4.7. The Concept of Tissue Micro-Instability and a Revised Definition of the OP

Our observations provide a basis for a novel interpretive concept, which we term *tissue micro-instability*. This concept posits, as a testable hypothesis, that joint-level micro-instability may manifest at the tissue level through the specific morphological alterations we describe. We propose that tissue micro-instability could represent the translation of joint micro-instability into structural failure within the vulnerable subchondral compartments identified. These critical sites include the VEPLS-zone cortical plate, foci of residual cartilage in the VEPLS zone, and osteoporotic subchondral trabecular bone.

Based on our morphological observations, we hypothesize that this process could be driven by persistent external shear forces originating from joint instability. In this proposed scenario, such instability likely results from various factors, including age-related degeneration of the ligamentous apparatus and other soft-tissue alterations that generate pathological force vectors. We postulate that the continuous application of these horizontal forces may lead to tissue disintegration and subsequent formation of an osteochondral cap. This structure, in turn, could undergo sustained shearing micromotion in the horizontal plane, potentially promoting further progression of trabecular micro-fractures and continued OP growth. Within this hypothetical framework, osteophytosis would thus represent a state of persistently stimulated bone regeneration and periosteal reaction in response to ongoing mechanical disruption.

Based on the observed histopathological progression, we propose the following hypothetical sequence of events, framed within the concept of tissue micro-instability:

1. Pre-OP stage (characterized by cortical protrusion and peripheral trabecular fracture)
  - Joint micro-instability induces fractures at the cortical angle, affecting both its subchondral and condylar aspects. Concurrently, fractures develop in the trabecula closest to the cortical angle.
  - Under persistent mechanical stress and impaired reparative osteogenesis, the cortical angle protrudes and the trabecular fracture extends linearly toward the central compartment.
2. OP emergence
  - Wavelike deformation of the condylar cortical plate arises within the VEPLS zone, accompanied by advancement of the protrusion and the formation of a distinct OP elevated above the original condylar surface.
  - Continued OP growth exacerbates deformation of the condylar cortical plate, resulting in compression of perforating epiphyseal vessels.
3. OP maturation
  The OP undergoes continued growth through enchondral and periosteal ossification, accompanied by recurrent cortical plate fractures and progression of the linear trabecular fracture. These fractures likely result from persistent mechanical trauma and further stimulate OP development.
4. Mature OP stage
  The OP detaches from the VEPLS-zone cortical plate while maintaining connections to the subchondral cortical plate and trabecular bone. This stage marks stabilization of the regenerative process, evidenced by the appearance of red bone marrow islands within the OP. Complete loss of articular cartilage and widespread osteosclerosis of the subchondral trabecular bone accompany this final stage.

**Figure 7:**
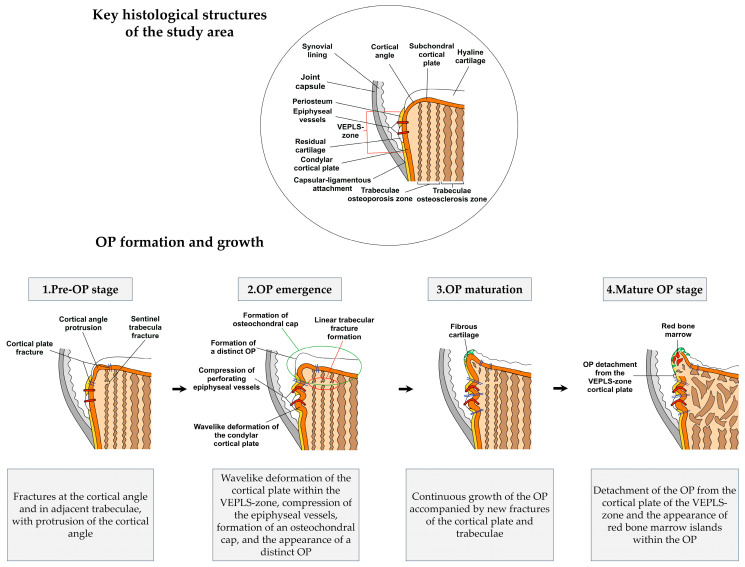
The scheme of histological events during OP formation and growth.

We graphically present the sequence of these events in Figure 7.

Thus, we define an OP as a pathological outgrowth originating from the condylar cortical plate and subchondral trabecular bone, resulting from chronic micro-fractures and compromised bone regeneration driven by tissue micro-instability.

### 4.8. Study Limitations

The interpretation of the results of this study should consider the following methodological constraints.

1. Sample size imbalance limiting evolutionary analysis

The primary limitation stems from the retrospective nature of cohort formation. The pre-OA group (Group 1, n = 5) and the early–moderate OA group (Group 2, n = 12) are substantially smaller than the late-stage OA cohort (Group 3, n = 68). This disproportion, which objectively reflects the extreme rarity of obtaining viable knee joint tissue from individuals without clinical OA, precludes robust statistical comparisons between the defined stages. Although the study’s aim was to describe a continuum of pathological changes rather than to perform direct intergroup comparisons, the limited sample size at the early disease phases may hinder the comprehensive capture of the full spectrum of possible initial pathological alterations and reduce the representativeness of the proposed descriptive evolutionary model.

2. Methodological limitations

This study employed a cross-sectional design. Tissues were analyzed at a single time point, which does not allow for the establishment of causal relationships or the tracking of the dynamic progression of OP formation and joint changes within the same individuals. Consequently, the proposed evolutionary trajectory is reconstructed from data obtained from different individuals, representing an inherent methodological limitation. Also, this study does not provide direct biomechanical measurements. Confirmation of the proposed concept of tissue micro-instability requires future experimental validation using appropriate in vivo models or biomechanical simulations designed to test the cause–effect relationship between shear forces and the described histopathological changes in the VEPLS zone and osteochondral cap.

3. Heterogeneous biomaterial sources and absence of radiographic data for key groups

Biomaterial for Groups 1 and 2 was obtained for reasons unrelated to knee joint pathology (malignancies, vascular catastrophe), and standardized radiographic examination of the knee joints was unavailable for these donors. This restricts the precise radiological characterization of the joint status at the time of tissue harvesting and creates a fundamental methodological discrepancy with Group 3, where the Kellgren–Lawrence classification was applied.

4. Single-center data

The study was conducted at a single clinical institution. Therefore, patient selection criteria and surgical practices may be specific to this center, potentially affecting the external validity of the findings.

Despite these limitations, this study provides valuable comparative histological insights across a uniquely broad spectrum of joint conditions—from presumably intact tissue to end-stage OA—which remains its principal strength.

## 5. Conclusions

We conducted a histological analysis of the OP microenvironment in human specimens spanning all OA stages, from earliest pre-chondropathy to late-stage disease. This comprehensive approach allowed us to reconstruct a potential developmental sequence for OPs and offers new insights into their histology and that of the subchondral bone in relation to OA progression.

Our findings suggest the involvement of bone structures in the tibial condyle’s vertical wall (VEPLS zone)—an area not previously emphasized as pathomorphologically significant—in OP formation. Drawing on these morphological observations, we introduce the concept of tissue micro-instability as a novel interpretive framework for considering OP pathogenesis, and propose revised working definitions for both OP and subchondral bone that are consistent with this concept. This framework appears to align more closely with a biomechanical model of OA pathogenesis than with a predominantly cartilage-centered one. It may therefore open avenues for future research into OA prevention and treatment, suggesting that osteophytosis could be viewed not only as a radiographic hallmark of disease severity but also as a potential indicator of ongoing mechanical dysregulation.

A key limitation of this study is its exclusively morphological basis; consequently, the tissue micro-instability hypothesis remains a conceptual model awaiting experimental biomechanical validation.

## Figures and Tables

**Table 1:** Histomorphometric analysis of cartilage and bone samples among the three groups. Statistical analysis of intergroup differences was performed using one-way ANOVA or Kruskal–Wallis tests with multiple comparisons. *p*-values ≤ 0.05 were considered statistically significant.

Morphological Parameters	Group 1	Group 2	Group 3	*p*-Value
load area, scores	1.0 [1.0; 1.5]	2.0 [1.0; 2.0]	2.0 [1.0; 2.0]	0.057 Gr.1 vs. Gr. 2: 0.046
height of cartilage, µm	2671.0 ± 1051.0	2914.0 ± 895.8	2219.0 ± 1125.0	0.042 Gr.2 vs. Gr. 3: 0.035
glycosaminoglycan content, scores	1.4 ± 0.5	2.1 ± 0.4	2.2 ± 0.9	0.022 Gr.1 vs. Gr. 2: 0.040 Gr.1 vs. Gr. 3: 0.019
diameter of the OP, µm	340.2 ± 334.6	2924.0 ± 1602.0	3899.0 ± 2089.0	<0.0001 Gr.1 vs. Gr. 2: 0.001 Gr.1 vs. Gr. 3: <0.0001
Cortical Plate Fracture Score				
subchondral zone, scores	1.0 [1.0; 2.0]	2.0 [1.0; 2.0]	2.0 [1.0; 2.0]	0.172
VEPLS zone, scores	1.0 [1.0; 1.0]	2.0 [1.0; 2.0]	2.0 [1.0; 2.0]	0.001 Gr.1 vs. Gr. 2: 0.002 Gr.1 vs. Gr. 3: 0.001
Subchondral Bone Thickness				
cortical plate, µm	286.0 ± 160.5	286.8 ± 153.6	344.1 ± 313.4	0.637
trabeculae, µm	143.7 ± 38.8	117.1 ± 36.4	140.8 ± 86.8	0.357
OP Microstructure Thickness				
subchondral cortical plate, µm	69.8 ± 75.0	149.7 ± 112.4	157.9 ± 131.4	0.142
trabeculae, µm	56.3 ± 66.3	107.4 ± 46.3	106.4 ± 36.7	0.010 Gr.1 vs. Gr. 2: 0.013 Gr.1 vs. Gr. 3: 0.011

## Data Availability

The data presented in this study are available upon request from the corresponding author.

## Supplementary Materials

The following supporting information can be downloaded at: https://www.mdpi.com/article/10.3390/biomedicines14020283/s1, Figure S1: Pearson correlation analysis.

## Abbreviations

The following abbreviations are used in this manuscript:

OP	Osteophyte
OA	Osteoarthritis
VEPLS zone	A special anatomical area where osteophyte develops
