# Supplementary figures and images for: A Novel Concept of Tissue Micro-Instability as the Underlying Mechanism of Osteophytosis in Human Knee Osteoarthritis
Source: Biomedicines. 2026 Jan 27;14(2):283. doi: 10.3390/biomedicines14020283 (PMC12938826; doi:10.3390/biomedicines14020283)

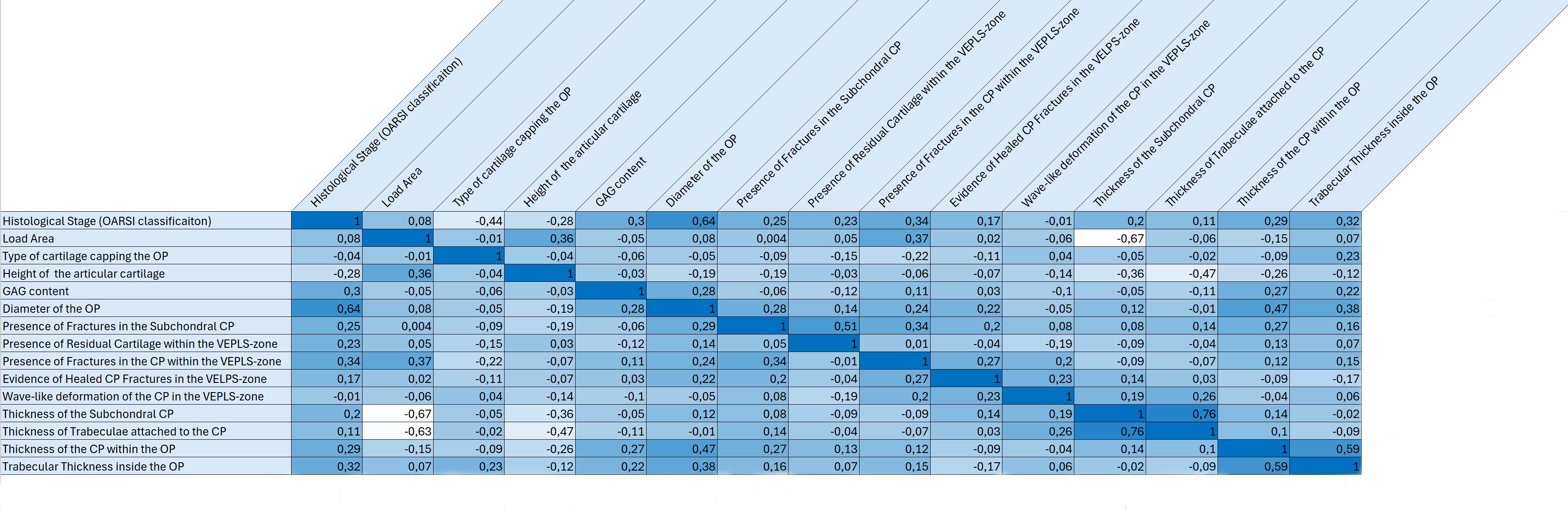

Supplement: Supplementary file 1 [file biomedicines-14-00283-s001.zip › biomedicines-4034448-supplementary.jpg]
